# Serotypes, virulence genes, and PFGE profiles of *Escherichia coli *isolated from pigs with postweaning diarrhoea in Slovakia

**DOI:** 10.1186/1746-6148-2-10

**Published:** 2006-03-20

**Authors:** Hung Vu Khac, Emil Holoda, Emil Pilipcinec, Miguel Blanco, Jesús E Blanco, Azucena Mora, Ghizlane Dahbi, Cecilia López, Enrique A González, Jorge Blanco

**Affiliations:** 1 Institute of Microbiology and Immunology, Department of Food Hygiene and Technology, University of Veterinary Medicine, Komenskeho 73, Slovakia; 2 Laboratorio de Referencia de *E. coli*, Departamento de Microbioloxía e Parasitoloxía, Facultade de Veterinaria, Universidade de Santiago de Compostela, 27002 Lugo, Spain; 3 Department of Bacteriology, Central Vietnam Veterinary Institute, km4 Dong De street, NhaTrang, Vietnam

## Abstract

**Background:**

Postweaning diarrhoea (PWD) in pigs is usually the main infectious problem of large-scale farms and is responsible for significant losses worldwide. The disease is caused mainly by enterotoxigenic *E. coli *(ETEC) and Shiga-toxin producing *E. coli *(STEC). In this study a total of 101 *E. coli *isolated from pigs with PWD in Slovakia were characterized using phenotypic and genotypic methods.

**Results:**

These 101 isolates belonged to 40 O:H serotypes. However, 57% of the isolates belonged to only six serotypes (O9:H51, O147:H-, O149:H10, O163:H-, ONT:H-, and ONT:H4), including two new serotypes (O163:H- and ONT:H4) not previously found among porcine ETEC and STEC isolated in other countries. Genes for EAST1, STb, STa, LT and Stx2e toxins were identified in 64%, 46%, 26%, 20%, and 5% of isolates, respectively. PCR showed that 35% of isolates carried genes for F18 colonization factor, and further analyzed by restriction endonuclease revealed that all of them were F18ac. Genes for F4 (K88), F6 (P987), F17, F5 (K99), F41, and intimin (*eae *gene) adhesins were detected in 19 %, 5%, 3%, 0.9%, 0.9%, and 0.9% of the isolates, respectively. The study of genetic diversity, carried out by PFGE of 46 representative ETEC and STEC isolates, revealed 36 distinct restriction profiles clustered in eight groups. Isolates of the same serotype were placed together in the dendrogram, but high degree of polymorphism among certain serotypes was detected.

**Conclusion:**

Seropathotype O149:H10 LT/STb/EAST1/F4 (14 isolates) was the most commonly detected followed by O163:H- EAST1/F18 (six isolates), and ONT:H4 STa/STb/Stx2e/F18 (five isolates). Interestingly, this study shows that two new serotypes (O163:H- and ONT:H4) have emerged as pig pathogens in Slovakia. Furthermore, our results show that there is a high genetic variation mainly among ETEC of O149:H10 serotype.

## Background

Postweaning diarrhoea (PWD) is usually the main infectious problem of large-scale farms and is responsible for significant losses worldwide [1,2]. The disease is caused mainly by enterotoxigenic *E. coli *(ETEC), and Shiga-toxin producing *E. coli *(STEC), also called verotoxin-producing *E. coli *(VTEC) [2-7]. Porcine pathogenic *E. coli *involved in PWD typically belong to serogroups O8, O138, O139, O141, O147, O149 and O157, of which O149 seems to be the predominant serogroup in most countries [1,5,7,8]. ETEC can cause severe diarrhoea in newborn and weaned piglets by the production of heat-labile enterotoxin (LT) and/or heat-stable enterotoxins (STa or STb). These enterotoxins are extracellular proteins or peptides, which are able to cause diarrhoea by changing the water and electrolyte balance of the small intestine [5]. Porcine STEC produce the edema verotoxin (VTe), also named Shiga toxin 2e (Stx2e), which damages the vascular endothelium of the small intestine, subcutis and brain and ultimately leads to subcutanneous edema and neurological disorders [9]. ETEC and STEC implicated in PWD in pigs most frequently produce either the F4 (K88) or F18 fimbrial adhesins [10,11]. Two variants of the F18 fimbriae exist: F18ab (F107) and F18ac (2134P) [11,12]. F18ac is associated with diarrhoea while F18ab is involved in edema disease [11]. In addition to F4 (K88) and F18, other fimbrial colonization antigens such as F5 (K99), F6 (P987), and F41 have also been associated with postweaning diarrhoea, but less frequently [4,13-15].

Porcine attaching and effacing *E. coli *(AEEC) induce intestinal lesions similar to those produced by enteropathogenic *E. coli *(EPEC) in humans. These *E. coli *carry *eae *gene encoding a 94 kDa outer membrane protein (intimin) which is responsible for intimate attachment to epithelial cells. However, the pathogenic significance of porcine *eae-*positive isolates in weaned pigs is unclear [16,17]. A new category of the diarrhoeagenic *E. coli *family, named enteroaggregative *E. coli *(EAEC), has been recognized. EAEC elaborate a low-molecular-weight, partially heat-stable, plasmid-encoded enterotoxin named enteroaggregative *E. coli *heat-stable enterotoxin 1 (EAST1). The gene (*astA*) encoding the production of EAST1 has been detected in several groups of diarrhoeagenic *E. coli *(EAEC, EPEC, ETEC, and STEC) isolated from humans and from pigs. The pathogenic significance of EAST1 in diarrhoea in pigs is not known [7,8,18-20].

Although, PWD is frequently observed in Slovakia, there is a lack of information about the prevalence of serogroups, serotypes, and virulence factors of porcine pathogenic *E. coli*. Thus, the aim of this study was to determine the distribution of serogroups, serotypes, and virulence genes, and to study the genetic relatedness among *E. coli *isolated from pigs with PWD. This is the first study in Slovak Republic of a large collection of pathogenic *E. coli *isolated from PWD.

## Results

### Serogroups and serotypes

The 101 porcine isolates belonged to 24 O serogroups and 40 O:H serotypes. However, 54% were of one of these eight serogroups (O8, O9, O45, O54, O141, O147, O149, and O163) and 57% of the isolates belonged to only six serotypes, including two new (O163:H- and ONT:H4) serotypes not previously found among porcine pathogenic *E. coli*. The most common serotypes were: O149:H10 (16 isolates), ONT:H- (13 isolates), O163:H- (11 isolates), O9:H51 (nine isolates), ONT:H4 (five isolates), and O147:H- (four isolates) (Table 1).

### Toxin genes

Amplification of the toxin genes by PCR showed that 77% of isolates possessed genes for production of five types of toxins: LT, STa, STb, Stx2e, and EAST1. The gene encoding for EAST1 toxin (65 isolates) was the most prevalent, followed by the STb (47 isolates), STa (27 isolates), and LT (20 isolates) genes. The Stx2e gene was detected in five isolates, which also carried genes for STa and STb (Table 1). Genes encoding Stx1, Stx1c, Stx1d, Stx2, Stx2c, Stx2d, and Stx2g toxins were not detected in any of the 101 porcine isolates studied.

### Adhesin genes

The PCR analysis of all 101 isolates of *E. coli *showed that 61 (60%) carried at least one fimbrial or intimin gene. The most prevalent fimbrial adhesin was F18, detected in 35 isolates. Analysis by restriction endonucleases of PCR F18-positive products revealed that all 35 F18-positive isolates showed the F18ac variant. Three of these 35 F18 isolates were also positive for either F4 or F17 genes. The gene encoding F4 was identified in 19 isolates. F6, F5, F41, and F17 genes were detected in five, one, one, and three isolates, respectively. The *eae *gene (intimin type β1) was detected in only one isolate (0.9%) of serotype O45:H- (Table 1).

Of the 19 *E. coli *F4-positive, 15 isolates belonged to O149:H10 serotype. The remaining four isolates belonged to O8:H19, O8:HNT, O118:H9, and ONT:H19. The F18 isolates were distributed in a wide range of serotypes, however, 29 of 35 isolates belonged to four predominant including O163:H- (11 isolates), ONT:H- (nine isolates), ONT:H4 (five isolates), and O147:H- (four isolates). Also F6 isolates were widespread among serotypes. Isolates carrying F17 gene belonged to O141:H25, O147:H-, and ONT:H- serotypes, and the isolate carrying both F5 and F41 genes belonged to ONT:H9 serotype.

Most isolates showing genes for fimbrial adhesins also possessed genes for toxin production, and the most common associations were: LT/STb/EAST1/F4 (18 isolates), EAST1/F18 (13 isolates), STa/STb/F18 (seven isolates), STa/STb/EAST1/F18 (five isolates), STa/STb/Stx2e/F18 (five isolates), STa/STb/EAST1/F6 (five isolates), and STa/STb/EAST1/F17/F18 (two isolates).

### Seropathotypes

Although the 101 porcine E. coli isolates belonged to 57 different seropathotypes (association between serotypes and virulence genes), only seven accounted for 39% of isolates. Seropathotype O149:H10 LT/STb/EAST1/F4 (14 isolates) was the most common, followed by O163:H- EAST1/F18 (six isolates), and ONT:H4 STa/STb/Stx2e/F18 (five isolates) (Table 1).

### Haemolytic activity

Haemolytic activity on blood agar plates was detected in 72 (71%) of the 101 *E. coli *isolates. All F4 and F18 isolates were haemolytic. Regarding the serotypes, all isolates of O149:H10, O163:H- and ONT:H4 serotypes were haemolytic (Table 1).

### Macrorestriction fragment analysis by pulsed-field gel electrophoresis (PFGE)

**Figure 1:**
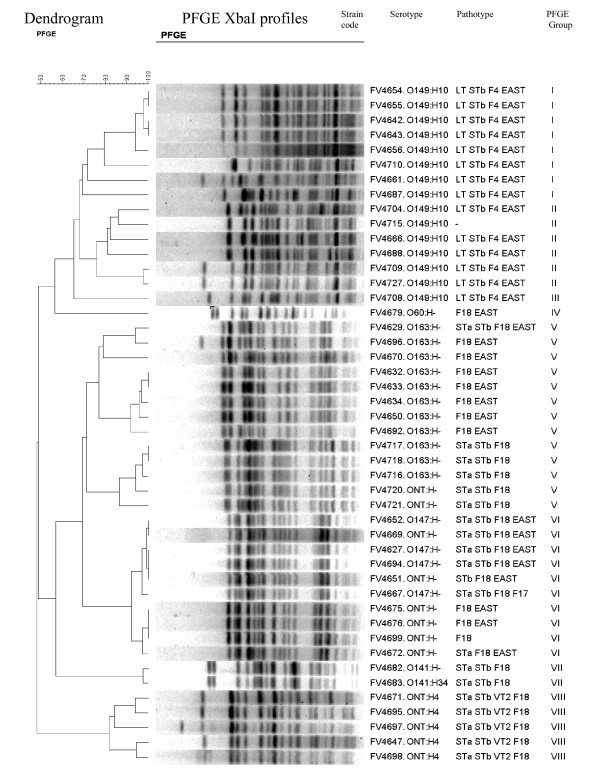
Dendrogram generated by Bionumeric software, showing distance calculated by the Dice similarity index of PFGE XbaI profiles among 46 strains isolated from PWD pigs. The degree of similarity (%) is shown on the scale.

A representative group of 46 isolates (45 ETEC and/or STEC) were selected to be analyzed by PFGE: O149:H10 (15 isolates), O163:H- (11 isolates), O147:H- (four isolates), ONT:H- (eight isolates), ONT:H4 (five isolates), O141 (two isolates), and O60:H- (one isolate). The study revealed 36 distinct restriction profiles, considering as significative a difference of a single band (Fig. 1). In the dendrogram produced by the UPGMA algorithm, the isolates were clustered in eight groups (I to VIII; 1 to 13 isolates per group) of 70% similarity according to the Dice similarity index, with 35 isolates clustering in nine subgroups of closely related (similarity > 85%) PFGE profiles. *E. coli *isolates of the same serotype were placed together in the dendrogram, but high degree of polymorphism among certain serotypes was detected. Thus, the 15 O149:H10 isolates were clustered in three groups (I-III, 70% similarity) with only three small subgroups of closely related profiles (similarity > 85%; five, two and two isolates, each). Genetic distance among O149:H10 isolates was as considerable as similarity < 66%. Group V clustered 13 isolates (all 11 O163:H- and two ONT:H- isolates; similarity > 71%) with three subgroups (two of them clustering five isolates each, similarity > 85%). Curiously, the highest homogeneity (similarity > 92%) was observed among a group of 10 isolates (group VI) belonging to serotypes O147:H- (four isolates) and ONT:H- (six isolates). *E. coli *isolates of serotypes O141:H- and O141:H34 were clustered in group VII (similarity > 97%). And all five isolates of serotype ONT:H4 clustered in group VIII showing a similarity > 81%.

## Discussion

It is widely accepted that specific serotypes and pathotypes of ETEC and STEC are responsible for the major part of PWD in piglets. However, the distribution and frequencies of serotypes and pathotypes can vary considerably from region to region and over time in a given region. The majority of the virulence factors are controlled by transferable genetic elements (plasmids and trasposons) and thus, common pathogenic seropathotypes may be replaced by previously uncommon types emerging as new pig pathogenic *E. coli*.

This is the first study in Slovak Republic of a large collection of pathogenic *E. coli *isolated from PWD. In the present study, although 101 isolates belonged to 40 different O:H serotypes, more than a half of ETEC and STEC belonged to only five serotypes: O147:H-, O149:H10, O163:H-, ONT:H-, and ONT:H4. Most isolates of these five serotypes possessed either the F4 or the F18 genes. ETEC of O147:H-, O149:H10 and ONT:H- serotypes are also frequently detected in pigs from other countries, especially O149:H10 [5,8]. The seropathotype O149:H10 LT/STb/EAST1/F4 (14 isolates) was the most prevalent in the present study, and the reason for its predominance is not known. A possible explanation could be that the virulence factor association of these isolates makes them especially adapted to propagation in swine populations and their enviroment [8].

The main discovery of this study was the identification of two new serotypes (O163:H- and ONT:H4) not previously detected among porcine ETEC and STEC isolated in other geographical zones. All 11 isolates of O163: H- serotype harbored the F18ac fimbriae gene and five of them were positives for STa and STb enterotoxins. Interestingly, all five Stx2e-positive isolates identified in the current study belonged to the new serotype ONT:H4. In previous studies the Stx2e production was associated mainly with O138:H14, O138:H-, O139:H1, O141:H4, O147:H6 and O157:H19 serotypes [5,12,21]. Thus, this study reports two new serotypes (O163:H- and ONT:H4) emerging as pig pathogens in Slovakia.

Although haemolysin does not seem to play an essential role in the virulence of porcine ETEC and STEC, most of the typical PWD *E. coli *are haemolytic [22]. In this study, 71% of isolates were haemolytic, and 66 isolates of those 72 possessed other virulence genes. Furthermore, all 32 isolates belonging to the three seroypes (O149:H10, O163:H- and ONT:H-) most frequently detected in this study showed haemolytic activity. Thus, the haemolytic activity is a very good marker for pig pathogenic *E. coli*.

F4 and F18 are the most important fimbrial adhesins of ETEC and STEC causing PWD [10,11]. In the present study using PCR analysis, it was shown that 35% of *E. coli *isolated from PWD carried genes for F18 colonization antigen. Our findings are in accordance with those of others [1,7,10,23,24]. Based on the studies of Rippinger *et al*. [25], the F18-family of fimbriae were divided into two variants F18ab and F18ac. The *E. coli *expressing F18ab cause edema disease, whereas the isolates with F18ac cause PWD [11]. In the present study, after digestion of PCR products of F18 isolates, all 35 F18-positive showed the F18ac variant, and more than a half of these 35 isolates belonged to O141, O147 and O163 serogroups. We found that 19 (19%) of 101 isolates from pigs with PWD carried the F4 gene. Several studies have demonstrated that the O serogroups associated with the fimbria F4 are mainly: O8, O149, and O157 [1,5,7,13,15]. Our results confirm these findings as 17 of 19 of Slovak F4 isolates belonged to O8 and O149 serogroups.

Zhu *et al*. [16] demonstrated that the *eae *gene is associated with A/E activity of O45 *E. coli *isolated from swine PWD. However, the AEEC are less commonly associated with PWD than ETEC. In the present study, we found only one O45:H- isolate (0.9%) positive for the *eae *gene. In accordance with our results, Frydendahl [7] and Osek [26] also found only 1% (3 of 219) and 3% (6 of 224) of Danish and Polish PWD isolates carrying the *eae *gene, respectively. F17-producing *E. coli *are commonly isolated from calves with or without diarrhoea [27]. In this study, the F17 gene was detected in three *E. coli*, two of which were also positive for F18, enterotoxins, and EAST1 toxin. Similarly, Osek [6] found that only four (1%) of 372 isolates from PWD in Poland were positive for F17.

The role of EAST1 toxin in swine colibacillosis has not been demonstrated, however, the gene encoding EAST1 toxin is commonly found in isolates associated with PWD [7,8,19,20]. Our results confirm these observations as we found that 65 of 101 isolates harbored *astA *gene and all F4 isolates were *astA *positive. The high frequency of the *astA *gene suggests the necessity of further studies to investigate the significance of this toxin in porcine PWD.

Genotyping methods such as multilocus enzyme electrophoresis (MLEE) and pulsed-field gel electrophoresis (PFGE) have been used for differentiation and epidemiological characterization of *E. coli *isolated from pigs with PWD and edema disease. PFGE is a powerfull tool to reveal inter- and intra-serotype specific genetic differences among porcine pathogenic *E. coli *[12,28,29]. However, there are few studies reporting genetic relatedness of *E. coli *isolated from diarrhoea in pigs. Osek [29] used the PFGE technique to analyze 82 *E. coli *from pigs with PWD, isolated from geographically separated farms in the western part of Poland. The 82 isolates belonging to four serogroups (O138, O139, O141, and O149) showed 13 different PFGE profiles and although a high degree of polymorphism among different serotypes was observed, isolates belonging to the same serological group showed a close relationship. Thus, the 25 isolates of serotype O149:K91 generated only two PFGE types. In our study, a representative group of 46 ETEC and STEC isolates revealed 36 distinct restriction profiles. Although isolates of the same serotype were placed together in the dendrogram, high degree of polymorphism among certain serotypes was detected. Thus, 13 distinct PFGE profiles resulted from 15 O149:H10 isolates analyzed, in spite of the fact that 14 *E. coli *of those 15 carried the same virulence genes (LT/ STb/EAST1/F4). Similarly results have been found in Spain among isolates of the most prevalent serotype (O157:H- LT/STb/F4) nowadays (unpublished data). Further studies are necessary to know if some of these clusters have appeared recently, and if so, analyze its evolution, as well as if there is any relationship with pathogenicity in farms.

## Conclusion

Our results indicate that in Slovakia, as described in other countries, pathogenic *E. coli *isolates from PWD belong to a restricted number of serotypes and pathotypes. The ETEC serotype O149:H10 seems to be predominant, but also two new serotypes (O163:H- and ONT:H4) not previously described in porcine ETEC and STEC isolated in other countries are common. The F18ac and F4 fimbriae were the most prevalent colonization factors detected in postweaning *E. coli *in Slovakia. Macrorestriction analysis showed that, although isolates of the same serotype and virulence markers mainly share the same PFGE group, there is a high genetic variation, especially among ETEC of O149:H10 serotype.

## Methods

### *E. coli *isolates

One hundred and one *E. coli *isolated from the same number of pigs with PWD were investigated in this study. The 101 post-weaning isolates came from 20 farms located in different parts of Slovakia. Five isolates were selected randomly from each farm, except one farm from Zemplinska Teplica, from which six isolates were collected. Of these 20 farms, nine farms were located in East part of Slovakia, seven farms in Central part of Slovakia, and the remaining four farms were in West part of Slovakia. The *E. coli *were isolated from the intestinal contents of carcasses of postweaning pigs with diarrhoea at the Department of Bacteriology (State Veterinary Institutes in Bratislava, Nitra, and Zvolen, Slovakia) and the Department of Food Hygiene and Technology (Institute of Microbiology and Immunology, University of Veterinary Medicine, Kosice, Slovakia) between 2001 and 2003. The fecal samples were plated onto MacConkey agar (Oxoid, UK) and the *E. coli *isolates were identified by standard biochemical procedures. After isolation, the *E. coli *were stored in Luria-Bertani broth containing 20% glycerol at -70°C for further characterization studies.

### Reference strains

The *E. coli *strains used as a control were: 298 (F4/K88), 329 (F5/K99), 318 (F6/987P), 320 (F41), 216 (F18 and Stx2e), 281 (LT), 256 (STa and STb), EDL933 (Stx1, Stx2 and *eae*-γ1), G491(F4/K88ac), P201 (F4/K88ad), 5138 (F18ab), 8813 (F18ac), 253KH85 (F17), 226KH85 (F17), 960205 (EAST1), 022206 (EAST1), and *E. coli *C600 (as negative control). Some of the control strains were kindly supplied by Dr. J. Osek (National Veterinary Research Institute, Pulawy, Poland), Dr. P. Alexa (Veterinary Research Institute, Brno, Czech Republic), Dr. P.F. Lintermans (Institut National de Recherches Veterinaires, Bruxelles, Belgium), and Dr. C. Chae (Department of Veterinary Pathology, College of Veterinary Medicine, Seoul National University, Republic of Korea).

### Serotyping

The determination of O and H antigens was carried out by the method described by Guinée *et al*. [30] employing all available O (O1–O181) and H (H1–H56) antisera in the LREC (Lugo). All antisera were obtained and absorbed with the corresponding cross-reacting antigens to remove the nonspecific agglutinins. The O antisera were produced in the LREC (Lugo, Spain) and the H antisera were obtained from the Statens Serum Institut (Copenhagen, Denmark).

### Haemolysin activity

The isolates were inoculated on blood agar base supplemented with 5% sheep blood (Oxoid, UK) and incubated at 37°C for 18h. β-Haemolysis was evident as a zone of lysis surrounding the bacterial growth.

### Detection of virulence genes by PCR

The polymerase chain reaction (PCR) for detection of toxins (LT, STa, STb, Stx1, Stx2, Stx2e, and EAST1) and adhesins (F4, F5, F6, F17, F18, F41, and eae) was carried out as described by Vu-Khac *et al. *[31] and Blanco *et al. *[32]. Base sequences and predicted sizes of the amplified products for the specific oligonucleotide primers used in this study are shown in Table 2[31-35]. Typing of *eae *(intimin) gene detected in one positive isolate identified in this study was carried out by PCR as described elsewhere [32].

### Digestion of PCR products with restriction endonuclease

After amplification, the PCR products of F18-positive isolates were digested with restriction enzyme *Ngo*MIV (formerly *Ngo*MI) to distinguish genes encoding F18ab and F18ac [33]. The master mix was prepared with a total of 15μ l volume containing 5μ l of PCR products (after purifying with Wizard PCR Preps [Promega]); 10μ l 1x MULTI-CORE™ buffer (Promega); and 1 unit of enzyme *Ngo*MIV. After incubation at 37°C for one hour, the DNA digestion was analyzed by electrophoresis in 2 % agarose gel.

### Pulsed-field gel electrophoresis

PFGE was performed in a CHEF MAPPER system (Bio-Rad, Hemel Hempstead, United Kingdom) at 14°C in 0.5XTBE by the Enternet proposed standard-protocol for PFGE [36]. Cleavage of the agarose-embedded DNA was achieved with 0.2–0.8 U/μ l Xbal (Roche) according to instructions of the manufacturer. Run times and pulse times were 2.20 to 54.0s for 22 h with linear ramping. PFGE was used to establish clonal relatedness and diversity among a representative group of 46 isolates. To perform the comparison of the PFGE pulsotypes, TIFF files were analyzed with BioNumerics software (Applied Maths, Sint-Martns-Latem, Belgium). Cluster analysis of the Dice similarity indices based on the unweighted pair group method using aritmetic averages (UPGMA) was done to generate a dendrogram describing the relationship among EPEC pulsotypes. A difference of at least one restriction fragment in the profiles was considered the criterion for discriminating between clones.

## Authors' contributions

H. Vu-Khac, E. Holoda, and E. Pilipcinec isolated the *E. coli *and performed the detection of virulence genes by PCR, M. Blanco, G. Dahbi and E. A. González also participated in the PCR study, J.E. Blanco did the serotyping of the isolates, A. Mora and C. López were responsible for the study of genetic diversity carried out by PFGE, and H. Vu-Khac, and J. Blanco designed the study and drafted the manuscript. All authors read, commented on and approved the final manuscript.

## Figures and Tables

**Table 1:** Serotypes, virulence genes, and hemolytic activity of 101 porcine *E. coli *isolates

Virulence genes	Total no. of isolates	Serotypes (No. of haemolytic isolates/Total no. of isolates)
LT STb EAST1 F4 F18	1	O149:H10 (1/1)
LT STb EAST1 F4	18	O8:H19 (1/1), O8:HNT (1/1), O118:H9 (1/1), O149:H10 (14/14), ONT:H19 (1/1)
LT STb EAST1	1	O8:H14 (0/1)
STa STb EAST1 F17 F18	2	O147:H- (1/1), ONT:H- (1/1)
STa STb EAST1 F18	5	O147:H- (3/3), O163:H- (2/2)
STa STb EAST1 F6	5	O2:H- (1/1), O9:H51 (2/2), O52:H45 (0/1), O64:H- (1/1)
STa STb	1	ONT:H- (0/1)
STa STb F18	7	O141:H- (1/1), O141:H34 (1/1), O163:H- (3/3), ONT:H- (2/2)
STa STb Stx2 F18	5	ONT:H4 (5/5)
STa EAST1 F18	1	ONT:H- (1/1)
STa EAST1 F5 F41	1	ONT:H9 (0/1)
STb EAST1 F18	1	ONT:H- (1/1)
STb EAST1	1	O141:H7 (0/1)
EAST1 F18	13	O9:H- (1/1), O60:H- (1/1), O163:H- (6/6), O175:H- (1/1), ONT:H- (4/4)
EAST1	16	O1:H34 (0/1), O9:H51 (4/5), O23:H15 (1/1), O45:H2 (0/2), O54:H- (3/3), O106:H15 (0/1), O123:H40 (0/1), O128:H28 (0/1), ONT:H- (1/1)
F17	1	O141:H25 (0/1)
*eae *(intimin type β1)	1	O45:H- (0/1)
None	21	O2:H1 (0/1), O3:H21 (1/1), O9:H51 (2/2), O20:H- (0/2), O23:H- (0/2), O23:H15 (1/1), O26:H28 (0/3), O71:H12 (0/1), O78:H11 (0/1), O84:H- (0/1), O141:H8 (0/1), O149:H10 (1/1), ONT:H- (0/2), ONT:H10 (0/1), ONT:HNT (0/1).

**Table 2:** Primer sequences and predicted size of amplification products

Target gene coding for virulence factors	Oligonucleotide sequences of primers	Amplified Products (bp)	Primer Coordinates	Accession number	Reference
LT	5'-ATT TAC GGC GTT ACT ATC CTC-3' 5'-TTT TGG TCT CGG TCA GAT ATG-3'	281	27–47 287–307	S60731	Osek *et al*. [35]
STa^a^	5'-TCC GTG AAA CAA CAT GAC GG-3' 5'-ATA ACA TCC AGC ACA GGC AG-3'	244	267–286^j ^492–510	M58746	Ojeniyi *et al*. [34]
STb	5'-GCC TAT GCA TCT ACA CAA TC-3' 5'-TGA GAA ATG GAC AAT GTC CG-3'	279	515–534 773–793	AY028790	Ojeniyi *et al*. [34]
Stx1all^b^	5'-CGC TGA ATG TCA TTC GCT CTG C-3' 5'-CGT GGT ATA GCT ACT GTC ACC-3'	302	113–134 394–414	M17358	Blanco *et al*. [32]
Stx2all^c^	5'-CTT CGG TAT CCT ATT CCC GG-3' 5'-CTG CTG TGA CAG TGA CAA AAC GC-3'	516	50–69 543–565	M59432	Blanco *et al*. [32]
Stx2e	5-ATG AAG AAG ATG TTT ATA GCG-3' 5'-TCA GTT AAA CTT CAC CTG GGC-3'	264	1176–1196 1419–1439	M36727	Osek *et al*. [35]
EAST1	5'-CCA TCA ACA CAG TAT ATC CGA-3' 5'-GGT CGC GAG TGA CGG CTT TGT-3'	111	2–24 94–114	S81691	Yamamoto and Nakazawa [18]
F4 (K88)^d^	5'-GCT GCA TCT GCT GCA TCT GGT ATG G-3' 5'-CCA CTG AGT GCT GGT AGT TAC AGC C-3'	792	31–54 798–822	M29374	This study
F5 (K99)	5'-TGC GAC TAC CAA TGC TTC TG-3' 5'-TAT CCA CCA TTA GAC GGA GC-3'	450	45–64 475–494	M35282	Ojeniyi *et al*. [34]
F6 (P987)	5'-TCT GCT CTT AAA GCT ACT GG-3' 5'-AAC TCC ACC GTT TGT ATC AG-3'	333	193–212 506–525	M35257	Ojeniyi *et al*. [34]
F17^e^	5'-GGG CTG ACA GAG GAG GTG GGG C-3' 5'-CCC GGC GAC AAC TTC ATC ACC GG-3'	411	289–310 677–699	AF055313	This study
F18^f^	5'-GTG AAA AGA CTA GTG TTT ATT TC-3' 5'-CTT GTA AGT AAC CGC GTA AGC-3'	510	1–23 490–510	M61713	Imberechts *et al*. [33]
F41	5'-GAG GGA CTT TCA TCT TTT AG-3' 5'-AGT CCA TTC CAT TTA TAG GC-3'	431	154–173 565–584	X14354	Ojeniyi *et al*. [34]
*eae*^g^	5'-GGA ACG GCA GAG GTT AAT CTG CAG-3' 5'-GGC GCT CAT CAT AGT CTT TC-3'	775	1441–1460 2193–2215	AF022236	Blanco *et al*. [32]

^a^Detects STaP and STaH variants.

^b^Detects Stx1 and the variants Stx1c and Stx1d.

^c^Detects Stx2 and the variants Stx2c, Stx2d, Stx2e, and Stx2g.

^d^Detects K88ab, K88ac, and K88ad.

^e^Detects F17a, F17b, F17c and F17d.

^f^Detects F18ab (F107) and F18ac (2134P).

^g^Detects all types of *eae *variants (Primer pair with homology to the 5' conserved region of *eae*).
